# Serotype Distribution and Antimicrobial Resistance of* Streptococcus pneumoniae* Isolates Causing Invasive and Noninvasive Pneumococcal Diseases in Korea from 2008 to 2014

**DOI:** 10.1155/2016/6950482

**Published:** 2016-05-30

**Authors:** Si Hyun Kim, Il Kwon Bae, Dongchul Park, Kyungmin Lee, Na Young Kim, Sae Am Song, Hye Ran Kim, Ga Won Jeon, Sang-Hwa Urm, Jeong Hwan Shin

**Affiliations:** ^1^Department of Laboratory Medicine, Inje University College of Medicine, Busan 47392, Republic of Korea; ^2^Paik Institute for Clinical Research, Inje University College of Medicine, Busan 47392, Republic of Korea; ^3^Department of Dental Hygiene, College of Health and Welfare, Silla University, Busan 46958, Republic of Korea; ^4^Department of Pediatrics, Inje University College of Medicine, Busan 47392, Republic of Korea; ^5^Preventive Medicine, Inje University College of Medicine, Busan 47392, Republic of Korea

## Abstract

*Introduction*.* Streptococcus pneumoniae* is an important pathogen with high morbidity and mortality rates. The aim of this study was to evaluate the distribution of common serotypes and antimicrobial susceptibility of* S. pneumoniae* in Korea.* Methods*. A total of 378 pneumococcal isolates were collected from 2008 through 2014. We analyzed the serotype and antimicrobial susceptibility for both invasive and noninvasive isolates.* Results*. Over the 7 years, 3 (13.5%), 35 (10.8%), 19A (9.0%), 19F (6.6%), 6A (6.1%), and 34 (5.6%) were common serotypes/serogroups. The vaccine coverage rates of PCV7, PCV10, PCV13, and PPSV23 were 21.4%, 23.3%, 51.9%, and 62.4% in all periods. The proportions of serotypes 19A and 19F decreased and nonvaccine serotypes increased between 2008 and 2010 and 2011 and 2014. Of 378* S. pneumoniae* isolates, 131 (34.7%) were multidrug resistant (MDR) and serotypes 19A and 19F were predominant. The resistance rate to levofloxacin was significantly increased (7.2%).* Conclusion*. We found changes of pneumococcal serotype and antimicrobial susceptibility during the 7 years after introduction of the first pneumococcal vaccine. It is important to continuously monitor pneumococcal serotypes and their susceptibilities.

## 1. Introduction

*Streptococcus pneumoniae* is one of the most common causes of pneumonia, sepsis, and meningitis and is the leading cause of morbidity and death worldwide in adults and children [1–3]. Ninety-two capsular serotypes of* S. pneumoniae* exist, and the prevalence of serotypes differs according to age, region, and time of the surveillance [4, 5]. The 92 serotypes differ in virulence; a minority of serotypes is involved in most of invasive pneumococcal diseases and antimicrobial resistances.

The 23-valent polysaccharide vaccine (PPV23) and a 7-valent pneumococcal conjugate vaccine (PCV7) were recommended for the elderly (≥65 years old) and children (≤5 years old), respectively. In Korea, PCV7, which protects against the important invasive serotypes (4, 6B, 9V, 14, 18C, 19F, and 23F), was introduced in 2003 for infants and young children. The introduction of PCV7 in the United States produced a decrease in both invasive and noninvasive pneumococcal diseases caused by these vaccine serotypes [6, 7]. However, use of PCV7 has led to changes in prevalent serotypes; it tended to increase the PCV7 nonvaccine serotypes, especially 19A, worldwide [8–10]. A second pneumococcal conjugate vaccine (PCV10 and PCV7 with serotypes 1, 5, and 7F added) and a 13-valent vaccine (PCV13 and PCV10 with serotypes 3, 6A, and 19A added) were introduced in Korea in 2010. Since May 2014, pneumococcal vaccination has been provided for free as a routine national vaccine program, including PCV10, PCV13, and PPSV23 in South Korea.

Since the first detection of* S. pneumoniae* with high resistance to penicillin and other antibiotics in 1977, high rates of antimicrobial resistance in* S. pneumoniae* have been a serious concern worldwide [11–13]. In Asian countries, beta-lactam and macrolide resistance are very high, and multidrug resistance (MDR) also is common [4, 14–16]. In 2008, the Clinical Laboratory and Standard Institute (CLSI) guideline changed the resistance breakpoint of nonmeningitis* S. pneumoniae* for penicillin from ≥2 *μ*g/mL to ≥8 *μ*g/mL [17]. Later, the resistance rate to penicillin decreased significantly; however, the high resistance rates to other antimicrobial agents have continued [18–20]. The aim of this study was to evaluate the changes in the prevalence of serotypes and their antimicrobial resistance during the past 7 years in Korea since the introduction of the vaccines.

## 2. Materials and Methods

### 2.1. Clinical Isolates

All 378* S. pneumoniae* isolates collected from patients at a tertiary-care hospital in Korea from January 2008 to June 2014 were included. The isolates were identified by colony morphology, gram staining, optochin susceptibility, and other biochemical reactions using VITEK2 system. All isolates were stored at −70°C using 10% skim-milk until use.

### 2.2. Serotyping

Serotyping was performed by capsular swelling (Quellung reaction) using Pneumotest antisera kit (Statens Serum Institut, Copenhagen, Denmark). For determining the serotype, pool antisera were used as recommended by the manufacturer. In order to determine additional serotypes, some factor antisera and serotype-specific polymerase chain reaction (PCR) recommended by the U.S. Centers for Disease Control and Prevention (CDC, http://www.cdc.gov/streplab/downloads/pcr-oligonucleotide-primers.pdf) were used [21]. Serotypes were classified into vaccine serotype (VT) and nonvaccine serotype (NVT). Vaccine serotype means a serotype included in PCV7 (serotypes 4, 6B, 9V, 14, 18C, 19F, and 23F), PCV10 (serotypes 1, 5, and 7F added to PCV7), PCV13 (serotypes 3, 6A, and 19A added to PCV10), and PPSV23 (serotypes 2, 8, 9N, 10A, 11A, 12F, 15B, 17F, 20, 22F, and 33F added to PCV13, except for 6A). Nonvaccine serotype is the serotype which is not covered by PCV7, PCV10, PCV13, and PPSV23.

### 2.3. Antimicrobial Susceptibility

Antimicrobial susceptibilities were determined using the Microscan system, and the susceptibility interpretive criteria were those published in the relevant guidelines of the Clinical and Laboratory Standards Institute (CLSI). Separate interpretive breakpoints were used to define the resistance to penicillin, cefepime, cefotaxime, and ceftriaxone for meningeal isolates. The following antimicrobial agents were tested: amoxicillin, azithromycin, cefaclor, cefepime, cefotaxime, ceftriaxone, cefuroxime, chloramphenicol, clindamycin, erythromycin, levofloxacin, meropenem, penicillin, tetracycline, sulfamethoxazole/trimethoprim (SXT), and vancomycin. The Food and Drug Administration defines multiresistance as resistance to two or more of the five classes of antibacterial agents represented by erythromycin, cefuroxime, SXT, penicillin, and tetracycline [22]. The resistance to cefotaxime and ceftriaxone was analyzed instead of that to cefuroxime and SXT. Macrolide resistance was defined by the erythromycin susceptibility test results.

We assessed differences in serotypes by age group, clinical specimens, surveillance periods, and resistance types. The data was analyzed with the software IBM SPSS version 22, using chi-square test.

## 3. Results

### 3.1. Characteristics of* S. pneumoniae* Isolates

The numbers of* S. pneumoniae* isolates by period are as follows: 198 isolates (52.4%) were obtained between 2008 and 2010 (period I) and 180 isolates (47.6%) between 2011 and 2014 (period II). Of the 378 isolates, 265 (70.1%) and 113 (29.9%) were from male and female patients, respectively. A majority (197; 52.1%) were obtained from the elderly (>65 years old) and 16 (4.2%) from children (≤5 years old). The mean age (±SD) of the patients was 60.8 ± 19 years (range 0–107 years). Of these isolates, 68 (18.0%) were invasive and 310 (82.0%) were noninvasive. The most common source of invasive isolates was blood (*N* = 60; 88.2%), followed by cerebrospinal fluid (*N* = 5; 7.4%), pleural fluid (*N* = 2; 1.5%), and ascitic fluid (*N* = 1; 1.5%). The sources of noninvasive isolates were the respiratory specimens (*N* = 281) including sputum and bronchial washing, pus (*N* = 26), and others (*N* = 3).

### 3.2. Distribution of Pneumococcal Serotypes

The most frequently isolated serotypes were 3 (13.5%), 35 (10.8%), 19A (9.0%), 19F (6.6%), 6A (6.1%), and 34 (5.6%), which together accounted for 51.6% of all isolates (Table 1). In children ≤ 5 years old, nine serotypes were identified, and 19A, 11A, and 23A were major serotypes, accounting for 37.5%, 12.5%, and 12.5% of the isolates, respectively. However, a diversity of serotypes was identified: more than 30 in the adults (≥65 years and 6–64 years old). However, serotypes 3 (14.2%, 13.9%) and 35 (11.2%, 10.9%) were most frequent.

The coverage rates of PCV7, PCV10, PCV13, and PPSV23 were 21.4%, 23.3%, 51.9%, and 62.4%, respectively. In children ≤ 5 years old, both PCV7 and PCV10 serotype coverages were same at 12.5%, whereas PCV13 covered 50.0%. For the elderly, PCV13 and PPV23 serotype coverages were 52.8% and 64.0%, respectively. Overall, vaccine serotypes were identified in 259 isolates (68.5%). Serotype 3 (19.7%) was the most common vaccine serotype, followed by 19A (13.1%), 19F (9.7%), and 6A (8.9%). Among nonvaccine serotypes, 35 (34.5%) was the most common, followed by 34 (17.6%), 6D (10.1%), and 6C (8.4%). We also compared the serotypes by dividing them as invasive and noninvasive isolates. For invasive isolates, the major serotypes were 3 (20.6%), 19A (10.3%), 35 (7.4%), 22F (5.9%), and 6A (5.9%), accounting for 50.0% of all invasive isolates. Serotype 19F was more common among noninvasive isolates (7.7%) than invasive isolates (1.5%). Ten serotypes were detected only among noninvasive isolates, accounting for 9.7%, and serotype 12F (4.4%) was detected only among invasive isolates (Table 2).

We analyzed the changes in serotypes over time by dividing the 7 years into two periods based on the introduction of PCV10 and PCV13 in Korea (period I: 2008–2010, *N* = 198, and period II: 2011–2014, *N* = 180) (Table 3). In period I, the major serotypes were 3 (12.1%), 19A (11.1%), 35 (9.1%), and 19F (8.6%), a total of 40.9%. In period II, serotypes 3, 35, 19A, and 6A were the most frequent and accounted for 41.1%. The number of nonvaccine serotypes increased to 37.2% in period II versus 26.3% in period I. In particular, serotype 35 showed a significant increase in the elderly. Among the vaccine serotypes, 19A, 19F, and 23F were decreased from 11.1% to 6.7%, 8.6% to 4.4%, and 4.5% to 1.1%, respectively. Serotype 3 was remarkably increased in the elderly (9.9% to 17.9%). However, it showed a decrease in patients 6–64 years old (15.5% to 11.8%). In children, the proportion of PCV13 serotypes decreased from 70% (*N* = 7) in period I to 16.7% (*N* = 1) in period II, which was attributable primarily to a decrease in serotype 19A.

### 3.3. Antimicrobial Resistance

The antimicrobial susceptibility of the* S. pneumoniae* is shown in Table 4. The resistance rates to erythromycin, tetracycline, azithromycin, cefaclor, cefuroxime, and clindamycin were high: 73.3%, 72.7%, 72.0%, 68.7%, 66.1%, and 56.9%, respectively.

The nonsusceptibility rate to penicillin was 26.8%, including 9.0% resistant and 17.8% intermediate. In children, the rates of penicillin resistance and intermediate susceptibility (20.0% and 46.7%, resp.) were significantly higher than in the elderly (8.5% and 17.1%) and younger adults (8.5% and 15.5%). In invasive* S. pneumoniae* isolates, the resistant and intermediate rates for penicillin were 6.7% and 16.7%, respectively. The rate of resistance of cefuroxime was high as 66.1%, and the resistance rates to cefotaxime and ceftriaxone were low, 4.1% and 4.7%, respectively. The resistance rate to levofloxacin was 7.2%, with the highest rate being 10.3% in the elderly. Also we found that the resistance rate to levofloxacin increased from 3.6% in period I to 11.7% in period II among all isolates (*p* value = 0.003).

Among the major serotypes, serotype 3 expressed low-level resistance to nine major antimicrobial agents. Serotypes 19A and 19F showed high resistance to most antimicrobial agents. For penicillin, serotypes 35, 19A, and 19F expressed high resistance (16.7%, 28.1%, and 19.0%, resp.) and serotypes 3 and 6B showed low resistance (2.4% and 7.7%). Serotypes 34, 11A, 6A, and 9V all were susceptible to penicillin. For levofloxacin, the resistance rates of serotypes 34, 5, 11A, and 9V were high: 15.8%, 15.8%, 23.1%, and 15.4%, respectively, whereas serotype 19A showed 100% susceptibility.

Of the total 378 isolates, 131 (34.7%) were multidrug resistant (MDR). The serotypes of most MDR* S. pneumoniae* isolates were vaccine serotypes (74.0%) consisting of 19A (20.6%), 19F (14.5%), 6A (7.6%), and so on. The rate of MDR* S. pneumoniae* was very high (68.8%) in children compared with those in the elderly (36.5%) and younger adults (29.1%). In period I, 78 isolates (39.4%) were identified as MDR; however, the prevalence of MDR* S. pneumoniae* was decreased to 53 isolates (29.4%) in period II when the vaccines were available. Although the resistance rate to penicillin was significantly decreased, to 3.6% from 13.3%, the resistance rate to levofloxacin was increased to 11.7% from 3.6%.

## 4. Discussion

This study describes the serotype and antimicrobial resistance of* S. pneumoniae* isolates in a tertiary-care hospital in Korea. We evaluated the change in the common serotypes and antimicrobial susceptibility patterns between 2008 and 2014. The distribution of serotypes was changed after PCV7 was introduced in Korea in 2003. In our study, the common serotypes were 3, 35, 19A, 19F, 6A, and 34 among all isolates, and the major invasive serotypes were 3, 19A, 35, 22F, and 6A. Compared with the previous report of Lee et al. [23] from 1996 to 2008, both serotypes 19F and 23F decreased and serotypes 3, 35, and 22F increased. This finding suggests that the proportion of PCV7 serotypes showed a decreasing trend, whereas there was an increase in the prevalence of non-PCV7 serotype between 1996–2008 and 2008–2014.

Our study from 2008 to 2014 showed that PCV7, PCV10, PCV13, and PPSV23 covered 21.4%, 23.3%, 51.9%, and 62.4%, respectively, of all isolates. The vaccine coverage of PCV7, PCV10, PCV13, and PPSV23 was reduced from 25.8%, 28.8%, 57.6%, and 68.2% to 16.7%, 17.2%, 45.6%, and 56.1% between period I and period II. Serotype 19A was significantly increased in children worldwide after the introduction of PCV7 [7, 24]. In Korea, Choi et al. reported that serotype 19A increased from 1996 to 2006 [25]. However, we also verified the change in the serotype's distribution after the introduction of PCV10 and PCV13, including the decline in the prevalence of serotype 19A from 11.1% in 2008–2010 to 6.7% in 2011–2014. On the other hand, serotypes 3 and 6A were slightly increased between 2008–2010 and 2011–2014.

We could see differences in the distribution of serotypes between invasive and noninvasive isolates. Ten serotypes (1, 8, 37, 11B, 15A, 17A, 17F, 18C, 23B, and 23F) were detected only among noninvasive isolates (7.1%), whereas serotype 12F was detected only as an invasive isolate. Also, the prevalence of serotype 19F was higher among noninvasive (7.7%) than invasive (1.5%) isolates, and serotype 22F was more common among invasive (5.9%) than noninvasive (1.9%) isolates. We also found a difference in the serotypes of* S. pneumoniae* by age. The common serotypes in children were 19A, 11A, and 23A, whereas serotypes 3 and 35 were predominant in adults. The lower occurrence of serotype 3 among the children has been reported in another study also [26]. A limitation of this study was the small numbers (only 4.2%) of isolates from children under 5 years who are the primary target age group for PCV vaccination.

Our findings confirm the previously reported high rates of resistance to macrolides such as erythromycin (73.3%), azithromycin (72.0%), and clindamycin (56.9%). The resistance rate to penicillin was higher (9.4%) in the invasive isolates in period I than in the ANSORP study [4] in Korea from 2009 to 2010 (0.3%). Also, the nonsusceptibility rate to penicillin was high in invasive isolates (23.4%) compared with the report of Park et al. [21] (10.7%) from 2009 to 2014. However, we found that the resistance rate to penicillin declined from 9.4% in period I (2008–2010) to 3.6% in period II (2011–2014) among invasive isolates. The interesting thing was that the intermediate resistance rate to penicillin increased slightly over the course of the study, from 15.6% to 17.9% among invasive isolates. We found that the resistance rate to levofloxacin increased significantly, from 3.6% in period I to 11.7% in period II among all isolates (*p* value = 0.003). Therefore, we need to continuously investigate the susceptibility rates for penicillin and levofloxacin.

The resistance rates in children were higher than those in other age groups, with 20% of isolates resistant to penicillin. Resistance to levofloxacin was not detected in children, whereas resistance was common in those more than 65 years old (10.3%). We do not know the exact reason why the resistant rates to levofloxacin have increased in the older age group. However, we guess the increasing use of levofloxacin is strongly associated with the higher resistance rates because the fluoroquinolone is commonly used as a choice of drug in adults for the respiratory tract infection while that is hardly used in young children. The resistance rates of noninvasive isolates were higher than those of invasive isolates; the rate of levofloxacin resistance was twice as high.

The proportion of MDR* S. pneumoniae* isolates, 34.7%, in all isolates and serotypes 19A, 19F, and 6A showed predominant serotypes in MDR* S. pneumoniae*. In children, the proportion of MDR* S. pneumoniae* was higher (68.8%) than that in other age groups (36.5% and 29.1% for younger adults and the elderly, resp.). The rate of MDR* S. pneumoniae* was decreased from 39.4% in 2008–2010 to 29.4% in 2011–2014. The reduction of MDR* S. pneumoniae* was associated with a decline in the proportion of 19A serotypes.

In this study, we evaluated the change of serotype distribution and antimicrobial susceptibility of all* S. pneumoniae* isolates in Korea over 7 years. We found a decrease of serotypes 19A and 19F and an increase in nonvaccine serotype 35. There were characteristic findings showing a high nonsusceptibility rate to penicillin in children and high resistance rates to levofloxacin. Therefore, we need continuous monitoring for changes of serotype and appropriate main antimicrobial agents.

## Figures and Tables

**Table 1:** Distribution of pneumococcal serotypes by patient's age group.

Serotype	Total (378) *N* (%)	Age group *N* (%)		
		≤5 years (*N* = 16)	≥65 years (*N* = 197)	6–64 years (*N* = 165)
3	51 (13.5)		28 (14.2)	23 (13.9)
35	41 (10.8)	1 (6.3)	22 (11.2)	18 (10.9)
19A	34 (9.0)	6 (37.5)	18 (9.1)	10 (6.1)
19F	25 (6.6)	1 (6.3)	15 (7.6)	9 (5.5)
6A	23 (6.1)		13 (6.6)	10 (6.1)
34	21 (5.6)	1 (6.3)	10 (5.1)	10 (6.1)
11A	15 (4.0)	2 (12.5)	9 (4.6)	4 (2.4)
6B	15 (4.0)		9 (4.6)	6 (3.6)
9V	15 (4.0)		8 (4.1)	7 (4.2)
15B	12 (3.2)		6 (3.0)	6 (3.6)
6D	12 (3.2)		8 (4.1)	4 (2.4)
23F	11 (2.9)	1 (6.3)	2 (1.0)	8 (4.8)
22F	10 (2.6)		7 (3.6)	3 (1.8)
6C	10 (2.6)	1 (6.3)	4 (2.0)	5 (3.0)
14	9 (2.4)		4 (2.0)	5 (3.0)
23A	9 (2.4)	2 (12.5)	2 (1.0)	5 (3.0)
7B	2 (0.5)	1 (6.3)	1 (0.5)	
Others^*∗*^	63 (16.7)		31 (15.7)	32 (19.4)

7-valent	81 (21.4)	2 (12.5)	41 (20.8)	38 (23.0)
10-valent	88 (23.3)	2 (12.5)	45 (22.8)	41 (24.8)
13-valent	196 (51.9)	8 (50.0)	104 (52.8)	84 (50.9)
23-valent	236 (62.4)	10 (62.5)	126 (64.0)	100 (60.6)

VTs	259 (68.5)	10 (62.5)	139 (70.6)	110 (66.7)
NVTs	119 (31.5)	6 (37.5)	58 (29.4)	55 (33.3)

VTs: vaccine serotypes; NVTs: nonvaccine serotypes.

^*∗*^Others include 15A, 13, 20, 33F, 10A, 4, 9N, 1, 16, 12F, 5, 24, 18C, 7F, 8, 37, 11B, 17A, 17F, and 23B.

**Table 2:** Comparison of pneumococcal serotypes between invasive and noninvasive organisms according to patient's age.

Serotype	Total *N* (%)	Invasive (age)			Noninvasive (age)		
		≤5	≥65	6–64	≤5	≥65	6–64
3	51 (13.5)		7	7		21	16
19A	34 (9.0)	1	3	3	5	15	7
35	41 (10.8)		1	4	1	21	14
22F	10 (2.6)		2	2		5	1
6A	23 (6.1)		1	3		12	7
11A	15 (4.0)		1	2	2	8	2
12F	3 (0.8)			3			
9V	15 (4.0)			3		8	4
4	4 (1.1)		1	1		1	1
16	3 (0.8)		1	1			1
20	6 (1.6)		1	1		1	3
34	21 (5.6)		2		1	8	10
23A	9 (2.4)	2				2	5
6D	12 (3.2)		1	1		7	3
5	2 (0.5)		1			1	
13	7 (1.9)			1		4	2
14	9 (2.4)		1			3	5
24	2 (0.5)			1		1	
10A	5 (1.3)			1		4	
15B	12 (3.2)			1		6	5
19F	25 (6.6)			1	1	15	8
33F	6 (1.6)		1			4	1
6B	15 (4.0)		1			8	6
6C	10 (2.6)		1		1	3	5
7B	2 (0.5)	1				1	
7F	2 (0.5)			1		1	
9N	4 (1.1)			1		1	2
1	3 (0.8)					1	2
8	1 (0.3)					1	
37	1 (0.3)						1
11B	1 (0.3)						1
15A	8 (2.1)					5	3
17A	1 (0.3)						1
17F	1 (0.3)						1
18C	2 (0.5)					1	1
23B	1 (0.3)						1
23F	11 (2.9)				1	2	8

Total	378	4	26	38	12	171	127

**Table 3:** Prevalence of common serotypes during two periods by patient's age.

Serotype	Total (378)		≤5 years (*N* = 16)		≥65 years (*N* = 197)		6–64 years (*N* = 165)	
	Period (P) I (198)	Period II (180)	P I (10)	P II (6)	P I (91)	P II (106)	P I (97)	P II (68)
3	24 (12.1)	27 (15.0)			9	19	15	8
34	12 (6.0)	9 (5.0)		1	6	4	6	4
35	18 (9.0)	23 (12.8)		1	8	14	10	8
19A	22 (11.1)	12 (6.7)	5	1	11	7	6	4
19F	17 (8.6)	8 (4.4)	1		10	5	6	3
23F	9 (4.5)	2 (1.1)	1		2		6	2
6A	11 (5.6)	12 (6.7)			4	9	7	3

7-valent	51 (25.8)	30 (16.7)	2		22	19	27	11
10-valent	57 (28.8)	31 (17.2)	2		26	19	29	12
13-valent	114 (57.6)	82 (45.6)	7	1	50	54	57	27
23-valent	135 (68.2)	101 (56.1)	8	2	63	63	64	36

VTs	146 (73.7)	113 (62.8)	8	2	67	72	71	39
NVTs	52 (26.3)	67 (37.2%)	2	4	24	34	26	29

VTs: vaccine serotypes; NVTs: nonvaccine serotypes.

**Table 4:** Resistance rates to antimicrobial agents by age.

Antibiotic	Total (378)			≤5 years (*N* = 16)			≥65 years (*N* = 197)			6–64 years (*N* = 165)		
	I	R	S	I	R	S	I	R	S	I	R	S
Amoxicillin	30 (8.2)	61 (16.8)	273 (75.0)	3	6	7	18	37	138	9	18	128
Azithromycin	11 (3.2)	244 (72.0)	84 (24.8)		13	2	7	126	39	4	105	43
Cefaclor	12 (3.7)	222 (68.7)	89 (27.6)		12	3	7	115	45	5	95	41
Cefepime	73 (22.0)	22 (6.6)	237 (71.4)	4	4	8	36	12	123	33	6	106
Cefotaxime	30 (9.4)	13 (4.1)	277 (86.6)	6	2	7	13	8	143	11	3	127
Ceftriaxone	33 (10.2)	15 (4.7)	274 (85.1)	6	3	6	13	10	142	14	2	126
Cefuroxime IV	11 (3.7)	199 (66.1)	91 (30.2)		12	2	8	101	46	3	86	43
Chloramphenicol	3 (0.9)	91 (26.7)	247 (72.4)		1	14	3	50	120		40	113
Clindamycin		193 (56.9)	146 (43.1)		13	2		106	71		74	73
Erythromycin	5 (1.4)	258 (73.3)	89 (25.3)		13	2	4	135	42	1	110	45
Levofloxacin	2 (0.6)	26 (7.2)	331 (92.2)			16	2	19	164		7	151
Meropenem	114 (32.9)	91 (26.2)	142 (40.9)	7	6	3	53	50	74	54	35	65
Penicillin IV	57 (17.8)	29 (9.0)	235 (73.2)	7	3	5	28	14	122	22	12	108
Tetracycline	5 (1.4)	256 (72.7)	91 (25.9)		13	2	2	130	49	3	113	40
Trimethoprim/sulfa	38 (10.3)	141 (38.3)	189 (51.4)	1	11	4	17	78	96	20	52	89
Vancomycin			352 (100)	****		15			181			156

I: intermediate; R: resistant; S: susceptible.
